# Willingness to report treatment-related symptoms of immunotherapy among patients with non-small cell lung cancer

**DOI:** 10.1007/s11136-021-02966-3

**Published:** 2021-08-12

**Authors:** Mona L. Martin, Helena Chung, Anna Rydén

**Affiliations:** 1 Evidera Patient Centered Research, Seattle, WA 98101 USA; 2 700 Wisconsin Avenue, Suite 1400, Bethesda, MD 20814 USA; 3 AstraZeneca Pharmaceuticals LP, Gaithersburg, MD 20878 USA; 4 AstraZeneca Gothenburg, Mölndal, Sweden

**Keywords:** Immunotherapy, Interviews, Non-small cell lung cancer, Qualitative research, Symptoms

## Abstract

**Purpose:**

Immunotherapy is an evolving therapeutic approach for non-small cell lung cancer (NSCLC). This study explored factors involved in patients’ perceptions about reporting or not reporting treatment-related symptoms experienced while undergoing immunotherapy.

**Methods:**

Patients receiving immunotherapy for NSCLC were recruited in the USA and Europe. Qualitative interviews were conducted to elicit treatment-related symptoms and explore patients’ reasons and motivations for either reporting or not reporting these to their medical teams. Interviews were audio-recorded, transcribed, and coded for qualitative analysis.

**Results:**

Sixty-six patients were interviewed (mean age: 62 years; 55% male; 91% with stage IV NSCLC). The most frequent symptoms that patients experienced but did not report were gastrointestinal (23% of patients), respiratory (17%), and energy related (12%). The most common reasons for not reporting symptoms included a perception that they were not severe enough, being unsure whether the experiences were side effects, and deciding that the experiences were expected and could be managed without assistance. Fear of having treatment discontinued was also mentioned but was not a prominent reason. The most common reasons for reporting symptoms were to ascertain if these were normal and expected, and to let the medical team know. Patients emphasized the importance of survival over treatment burden when balancing symptoms with treatment benefits.

**Conclusion:**

Patients have a range of reasons for not reporting their treatment-related symptoms when undergoing immunotherapy for NSCLC. Reasons are more strongly related to determination of the severity versus manageability of patients’ experiences of symptoms than they are to the fear of having treatment discontinued.

## Plain English summary

Immunotherapy is a new type of treatment that helps the body’s immune system to fight cancer. People with cancer who receive immunotherapy often experience symptoms because of their cancer and the treatment. If a person receiving immunotherapy has treatment-related symptoms it is important to let the doctor or medical team know. Many treatment-related symptoms improve if managed early on. Having symptoms under control helps patients to stay on their treatment. Researchers believe that many patients do not tell their doctors about their treatment-related symptoms. The reasons for this are unclear. One suggestion has been that people might worry about being taken off their treatment by their doctors if they mention their symptoms. In this study, we interviewed people who were receiving immunotherapy for non-small cell lung cancer (NSCLC) about why they decide to report or not report treatment-related symptoms to their doctor. Overall, 66 people from France, Spain, the UK, and the USA were interviewed. The most common reason people had for not reporting a symptom was thinking it was not important enough. In contrast, only a few people said that they did not report a symptom because they worried that their treatment would be stopped. The most common reason why people did report a symptom was that they wanted to know if it was normal and expected. Doctors need to communicate clearly and early to patients with NSCLC receiving immunotherapy which treatment-related symptoms to expect, how these might be managed, and when to reach out for help.

## Introduction

Little is known about what patients consider when deciding whether to report symptomatic, possible treatment side effects while undergoing therapy for cancer or other life-threatening diseases. There are a range of potential reasons why patients may decide not to report treatment-related symptoms. One reason commonly cited by healthcare providers is patients’ fear of being discontinued from a clinical trial [1, 2]. Other cited reasons include patients misunderstanding what information needs to be reported or having difficulty in recalling their symptoms [1, 2]. Little information is available directly from patients as to why they may decide not to report treatment-related symptoms. Patients with lung cancer participating in a roundtable discussion commented that they might underreport symptoms, side effects, and physical limitations during clinical trial participation owing to fear of it affecting their care while in the trial, including concern that they might be removed from the trial [3]. The different reasons patients might have for not reporting treatment-related symptoms and whether there are additional factors that affect their decisions to report or not report symptoms are not well understood.

Immunotherapy in oncology has shown promise in the treatment of non-small cell lung cancer (NSCLC), demonstrating improved overall survival rates compared with typical cytotoxic chemotherapies [4]. Immuno-oncology therapy has also been shown to affect other immune-mediated events, leading to dermatological, gastrointestinal, endocrine, and, more rarely, hematopoietic and urinary system side effects [5, 6]. With timely recognition and management, many of these side effects are reversible [6].

Understanding patients’ willingness and motivations to report immune-mediated adverse events and other potential side effects allows healthcare providers to institute programs to educate patients about realistic expectations with immunotherapy, as well as consider more proactive monitoring of side effects during treatment. Early reporting of treatment-related symptoms by patients is essential to enable their medical teams to assist in symptom management and lessen patients’ treatment burden. This type of care is critical in helping patients achieve desired treatment experiences and outcomes by improving their ability to remain on treatment.

The objective of this study was to identify different attitudes, values, and considerations involved in patients’ willingness and motivation to inform their medical teams about potential side effects experienced while undergoing immunotherapy for advanced NSCLC.

## Patients and methods

The methodological orientation underpinning the study was a qualitative content analysis by theme. Initial themes relating to study objectives and interview questions were used to start a coding framework, which was adjusted appropriately as more concepts and themes were identified in the interview transcripts.

### Study design and participants

This qualitative interview study recruited patients with NSCLC from France, Spain, the UK, and the USA. To be eligible, patients had to be aged 18 years or older, have a diagnosis of NSCLC, and either be currently receiving immunotherapy for NSCLC or have completed immunotherapy for NSCLC within the last 12 months. Eligible patients also had to meet at least two of the following self-reported characteristics indicating that their NSCLC was likely to be advanced: have multiple treatment experiences; be more than 1 year past their NSCLC diagnosis; have metastatic disease; have at least moderate symptoms; and/or have at least stage III NSCLC.

For the USA-based clinic sites, potential participants meeting the basic eligibility criteria were identified by chart review and were approached by site staff via telephone or in person during regular clinic visits. Eligible patients from a large, USA-based patient group, Patient Power, were identified by telephone screening of interested patients and self-report of eligibility criteria. Potential participants in Europe were identified by market research companies via social media or from marketing databases. Potential participants were also screened for a purposive sampling quota of those who were frequent users of social media and other patient support group activities and those who were not. This allowed comparison of results between those who might be influencing each other in the social media context to report their symptoms versus those who did not receive such encouragement because of low levels of interaction. Having some patients recruited from medical clinics and some patients from patient groups and market research lists allowed us to compare results by those who self-reported their eligibility and those confirmed by medical records. Having a sizeable qualitative sample from the USA and from European countries allowed us to compare results for any differences in willingness to report side effects that might be coming from a difference in healthcare systems or cultures.

All potential participants were informed about the study and were administered screening questions using the same institutional review board-approved script. If they were interested and had given their verbal informed consent, they were scheduled for an interview session and provided with a paper consent form to sign and return prior to the interview. There were no dropouts amongst patients who had agreed to participate.

The study was conducted at all sites in accordance with the Declaration of Helsinki ethical principles and with Good Clinical Practice, and the protocol and study forms and activities were approved by the Quorum institutional review board (Seattle, WA, USA). Market research companies used in Germany, France, and the UK were also compliant with the EphMRA code of conduct appropriate for research activities that do not use any medical information from the national healthcare system or from private doctors.

### Concept elicitation interviews

Patients were not expected to be able to attribute their experiences accurately to their disease or its treatment. Therefore, ‘treatment-related symptoms’ was used as collective terminology to capture patients’ experiences during treatment of their NSCLC.

A semi-structured interview guide with open-ended questions and follow-up probes was used to elicit patients’ perceived experiences of symptoms related to immunotherapy, to inquire whether patients reported their symptoms to their medical teams, and to determine what factors patients considered when deciding whether to report or not to report their symptoms. The interview guide was pilot tested via three to four mock interviews conducted during the interviewer training process between the interviewers. Patients were asked about their thought processes while deciding whether to report symptoms. Near the end of the interview, patients were asked to describe their thoughts about balancing possible treatment-related symptoms versus possible benefits of treatment. The corresponding text in the interview guide was: “What goes through your mind when you think about the balance between the possible side effects of your treatment and the possible benefits of the treatment?”.

Interviews were conducted via telephone by trained and experienced qualitative interviewers. The interviewers introduced themselves and provided an overview of the research and its aims. Each interview lasted between 60 and 90 min. Interviews were conducted in native language and were audio-recorded and transcribed for qualitative analysis. Non-English interview audio files were moved into English audio files by direct simultaneous interpretation. All transcripts were developed in English and coded using ATLAS.ti software to organize the assigned codes. Patients were reimbursed for their participation in the interviews.

### Analysis of qualitative data

An initial coding framework for organizing and grouping coded information was developed from the qualitative interview guide and was revised as needed when new concepts were identified in the interview transcripts. ATLAS.ti version 7.1.0 (ATLAS.ti Scientific Software Development GmbH, Berlin, Germany [7]) was used to assign codes that would organize the transcript data by similarity of content or themes.

The purposive sampling for high and low social media use, as well as the clinic versus self-report groupings of the study population, and the US versus European groupings, were all examined to identify any differences relative to those groupings in the willingness of patients to report their symptoms and side effects to their doctor. Symptoms that patients experienced but did not report were compared by each of these aspects.

Six interview transcripts were independently dual coded and assessed for inter-rater agreement, targeting 90% agreement or above [8]. Saturation of concept was evaluated by ordering transcripts chronologically into five groups (13–14 transcripts per group) based on interview completion date and comparing the codes newly identified in each subsequent grouping. Saturation of concept was considered to be reached when no new concepts were forthcoming [9].

## Results

### Patients

In total, 66 patients were interviewed: 12 in France, 9 in Spain, 15 in the UK, and 30 in the USA (social media-based recruitment: Washington, *n* = 15; clinic-based recruitment: Illinois, *n* = 8 and California, *n* = 7). The overall mean age was 62 years (range: 39–88 years), 55% of patients were men (Table 1), and 87% were White. Most patients (91%) reported having stage IV NSCLC, and 40% rated the severity of their NSCLC symptoms as moderate to very severe. All patients had received immunotherapy for their NSCLC, in accordance with study eligibility criteria, and 74% reported their type of treatment to be monotherapy.

**Table 1:** Demographic, health-related, and NSCLC-related characteristics

Characteristic	All patients (*N* = 66)
Age, years, mean (SD) [range]	61.6 (9.3) [39–88]
Male, *n* (%)	36 (54.5)
Patient-reported overall health, *n* (%)	
Excellent	4 (6.1)
Very good	20 (30.3)
Good	21 (31.8)
Fair	17 (25.8)
Poor	3 (4.5)
Unknown/missing	1 (1.5)
Time since NSCLC diagnosis, years, mean (SD) [range]	3.1 (2.2) [0.1–10.1]
Stage of NSCLC at diagnosis, *n* (%)	
I	2 (3.0)
II	4 (6.1)
III	15 (22.7)
IV	43 (65.2)
Unknown/missing	2 (3.0)
Current stage of NSCLC, *n* (%)	
I	1 (1.5)
II	1 (1.5)
III	2 (3.0)
IV	60 (91.0)
Unknown/missing	2 (3.0)
Metastases from lung (if known), *n* (%)	50 (75.8)
Cancer treatments received, *n* (%)	
Surgery	20 (30.3)
Radiation therapy	37 (56.1)
Chemotherapy	51 (77.3)
Targeted therapy	8 (12.1)
IO therapy	66 (100)
Type of IO therapy, *n* (%)	
Monotherapy	49 (74.2)
Combination therapy	9 (13.6)
Unknown/missing	8 (12.1)
Experienced side effects with IO therapy (if known), *n* (%)	32 (48.5)

*IO* immuno-oncology, *NSCLC* non-small cell lung cancer, *SD* standard deviation

### Data quality

#### Saturation of concept

Twenty-three concepts were identified from the qualitative interviews, covering expectations of treatment benefits and treatment-related symptoms, reasons for reporting or not reporting symptoms, coping strategies for dealing with treatment-related symptoms, types of interactions with the healthcare team, level of engagement with patient groups, and previous experience in clinical trials. Concept saturation was achieved by the third transcript group (approximately 42 interviews). Twenty-two concepts (96%) arose in the first group of interview transcripts and one additional concept (4%) arose in the second group.

#### Inter-rater agreement

Inter-rater agreement on the presence of a concept needing to be assigned a code ranged from 91 to 99% across six dual-coded transcripts. Agreement on the specific code that was assigned was between 96 and 100%.

### Predominance of concepts

During the interview discussions, the words ‘symptoms’ and ‘side effects’ were commonly used interchangeably. When asked to attribute their experiences either to their disease or to its treatment, patients reported thinking of respiratory experiences such as coughing, coughing up blood, wheezing, and shortness of breath as symptoms of their cancer. Most other experiences were attributed by patients to both their cancer and its treatment (e.g. fatigue) or solely to their cancer treatment (e.g. weight gain). Approximately half of patients (49%) reported that they experienced side effects during immunotherapy, and an additional 23% reported that they did not know if the symptoms they experienced were due to their cancer or to the effects of their treatment.

#### Symptoms not reported

There was minimal variation between patients in the USA and Europe, or between high and low users of social media, in terms of the symptoms that patients experienced and had reasons not to report (Table 2). The most common symptoms not reported were gastrointestinal, respiratory, and energy related. Patients who were high social media engagers seemed to have experienced more pain/discomfort than low engagers, but still did not report it.

**Table 2:** Treatment-related symptoms patients experienced and had reasons for not reporting

Symptom/side effect category	Geographical region		Social media use	
	USA (*n* = 30) *n* (%)	Europe (*n* = 36) *n* (%)	Low (*n* = 44) *n* (%)	High (*n* = 22) *n* (%)
Energy-related	4 (13)	4 (11)	5 (11)	3 (14)
Pain and discomfort	2 (7)	2 (6)	3 (7)	7 (32)
Respiratory	6 (20)	5 (14)	8 (18)	3 (14)
GI-related	5 (17)	10 (28)	10 (23)	5 (23)
Urinary/bladder-related	2 (7)	1 (3)	3 (7)	0 (0)
Systemic	2 (7)	3 (8)	4 (9)	4 (18)
Sleep disturbances	2 (7)	3 (8)	5 (11)	0 (0)
Ocular	1 (3)	1 (3)	1 (2)	1 (5)
Cognitive	1 (3)	3 (8)	4 (9)	0 (0)
Other^a^	10 (33)	12 (33)	14 (32)	8 (36)

*GI* gastrointestinal

^a^Other symptoms not reported in this table included: anemia, anxiety, bruising, drooling, hair changes, hair loss, inability to eat certain foods, itching, nail problems, neuropathy/numbness, range of motion issues, rash, ruined veins, sensitivity to sun, sexual issues, skin problems, swelling, unable to grip objects, vaginal bleeding, and vaginal dryness

#### Reasons for not reporting symptoms

Patients’ reasons for not reporting treatment-related symptoms are shown in Table 3, together with example quotations. The most frequently mentioned reason was the impression that the symptom was not sufficiently significant to warrant being reported (44% of patients interviewed). Other reasons included doubting whether the symptom being experienced was a side effect or not (16%), feeling that they could manage the symptoms themselves (12%), fear of being discontinued from treatment (12%), forgetfulness (8%), and thinking that the medical team would not be able to help (8%).

**Table 3:** Patient-reported reasons for not reporting treatment-related symptoms

Reason for not reporting symptoms	Patient expressions (*n* = 25) *n* (%)	Example patient quotations
Insignificant	11 (44)	“I guess thinking it’s just kind of trivial” “If it wasn’t bothering me much…I didn’t see any reason to report it” “If I’m not bothered by it…If I can live with it” “I felt as if it’s not important to report, it might just be normal”
Not sure what was going on	4 (16)	“…if I knew where it comes from…” “It was nothing very specific” “I didn’t really think of the medication at first, I didn’t know what was going on”
Can take care of it myself	3 (12)	“I thought I could take care of it myself” “Something feels trivial to me, that I can manage myself” “It might not be related to the pathology, so I may be able to find a solution on my own”
Fear of being taken off treatment	3 (12)	“Afraid of being switched to another cancer drug” “Fear of them taking me off [xx], because I don’t really want to stop because it’s holding everything steady right now”
Forgetfulness	2 (8)	“I forgot to mention it” “Forgetfulness is really the reason I wouldn’t report a side effect”
Healthcare team can’t do anything about it	2 (8)	“They can’t do anything about it…or it’s too personal” “I’m pretty sure there is nothing they can do about it”

#### Reasons for reporting symptoms

Patients’ reasons for reporting their symptoms are shown in Table 4, together with corresponding example quotations. The most common reasons related to wanting to know whether what was being experienced was normal or not (34% of patients interviewed). Patients explained that they wanted to know what was happening in their body and how to think about it in relation to their disease and their treatment. The second most common reason (20%) was wanting to provide the information their medical team needed to better monitor them and their reactions to the treatment. Other reasons included having been prompted by their medical team to watch for specific symptoms (15%), wanting to have some relief from the severity of the symptoms (12%), trying to find out if remedy was possible (12%), and wishing to mitigate the impact the symptoms were having on their life (7%).

**Table 4:** Patient-reported reasons for reporting treatment-related symptoms

Reason for reporting symptoms	Patient expressions (*n* = 41) *n* (%)	Example patient quotations
To find out what was going on; was it normal?	14 (34)	“Just because I would like to know if it’s normal or not normal from the chemo” “When I notice something different, I want to know why” “I want to make sure everything is going to be alright” “I would want further investigations or reassurance”
Because my healthcare team needs to know	8 (20)	“I just tell them, so they know what I am going through” “I want them to know I have these things so they can document it” “If anything at all is wrong with me, I tell them”
It was an identified side effect they were supposed to report if it happened	6 (15)	“They continually say ‘if you are feeling anything or you are sick, call right away’” “My duty to report and see if those are side effects of the medication” “If you don’t tell them the truth, they cannot help you”
The severity	5 (12)	“If it gives me problems, I would tell” “Extreme pain and discomfort” “The pain, I’m scared of pain”
To see if it could be fixed	5 (12)	“To find out what’s going on and get it fixed” “Looking for some relief” “To see if it can be stopped”
Impact on my life	3 (7)	“They are impacting my lifestyle” “How detrimental they are to your daily activities”

#### Balancing possible treatment-related symptoms with treatment benefits

Patients were asked to describe their thoughts on balancing possible treatment-related symptoms with possible treatment benefits (“What goes through your mind when you think about the balance between the possible side effects of your treatment and the possible benefits of the treatment?”). Several themes emerged. These were common across study sites and levels of social media engagement. Example quotations are shown in Table 5.

**Table 5:** Patient views about balancing treatment-related symptoms with treatment benefits

“[It’s] keeping me alive… I’d rather for it to be going slow with [treatment] than too fast without [treatment]”
“On [treatment] you take a risk of falling, losing your balance…but it’s either that or die with cancer”
“Simply the cancer that I have, I don’t know too many people that live from prostate cancer, so, yes, I want to survive”
“[My thinking was] let’s say that I have 6 months to live. And I go through this treatment and I’ve got another 6 months to live, but I’m so sick during those 6 months that it’s not worth…being alive, then I don’t want to do it”
“[They say] this could happen and that could happen…they don’t say it will. So, if it could happen, but if it doesn’t—it doesn’t. And that’s basically how I weighed in …I just realize that I have to go through what I’m going through because it’s going to make me better…”
“I'm hoping that the benefit is that it goes into remission and that I don’t have to worry about it anymore…hoping that we can get rid of it”
“I decided okay, the only way to find out is to try this and hope that it works out. It did not for my dad… And I thought, okay I owe it to my wife, my son, my friends”
“I think that if we have something that’s shown to be a good solution to a problem, that I would try it…and it depends on how severe the side effects would be”
“It also to me depends on what stage of a disease I’m in. If I’m totally bedridden on machines and the chance that some drug might give me six extra months, that’s not worth it to me… But if I’m in the normal life… there are drugs that have helped people live an extra 3, 5, 10 years or even go into remission and not have symptoms come back”
“The benefits far outweigh the side effects…way far outweigh…if I’d been diagnosed with stage III lung cancer 4 years ago, I’d be dead now [because the treatment wasn’t available]”
“The fact that I know that I got through it before and I know that I’m going to get through it now and I’m going to get better. So, I just hang in there and just go with it”
“The side effects it’s kind of irritating, but I can deal with it…I’m breathing on my own now. So, the chemo is coming along beautifully”
“Well I didn’t have any other treatments to compare it to, it was what we started with…I think it’s great. I can totally function again”
“Well, it depends probably what the side effects are, right, is it going to hamper me and what I’m trying to do, is it going to make me live longer, what are the benefits?”
“Well put this way, [treatment] is keeping me alive”
“Yes, I want to live so I have no choice but to take the treatment”
“…I was a little fearful about it…side effects…I just decided that was probably the best route to go and if that worked then it’s worth it”
“[I thought about potential side effects] only so much as you can imagine in your mind what they’re telling you what to expect. Until you’ve lived it, it’s just words on paper. You don’t know…what chemotherapy nausea is like…not like any nausea you’ve ever had before”
“Absolutely, I look at [side effects but] when you get a condition like cancer, I just put complete faith in the medical profession”
“I am not interested in the side effects at the start. I’m more interested in the treatment which is supposed to make me feel better. I judge the treatment basically, not the side effects”
“I just tell myself that I must not think about the side effects straight away [and do treatment to be healed]”
“I’m always told that there was a risk, I knew the risk. I was ready to take that risk, I didn’t really have a choice”
“I’ve got to go for it…I’m quite willing to try anything [despite side effects]…when they tell you that there’s activity again you’ve got to go for it…it’s not really an option”
“I’d made up my mind that it’s [beneficial] even in spite of the side effects…it’s pretty much a no-lose gamble”

Patient responses placed heavy emphasis on survival. Many patients cited survival as their main consideration and noted that treatment-related symptoms were to be expected. The strongest theme was that there was no choice but to try treatment regardless of treatment-related symptoms because of the dire alternatives that patients were facing.

Other themes included limitations in patients’ ability to tolerate treatment-related symptoms, and patients wanting to maintain their ability to function and have a better quality of life with the time they had left to live. Some patients noted that they needed to trust their doctors to make the right decisions regarding the treatments they were placed on.

## Discussion

In this qualitative study, we examined different aspects of patients’ willingness to inform their medical team about treatment-related symptoms of their immunotherapy for NSCLC. Using the terminology ‘treatment-related symptoms’ focuses on patients’ recognition of having a symptom or sensation and links it to the time they were receiving treatment.

A notable study finding was that the primary reason for not reporting treatment-related symptoms was because patients discounted the significance of the event, rather than a concern that their treatment would be discontinued. While the literature suggests that patients enrolled in clinical trials for lung cancer have a tendency to under-report symptoms and side effects for fear of being removed from the trial [3, 10], our results from these interviews with patients with NSCLC outside of the clinical trial setting suggested that while fear is a consideration for some, the primary reason for not reporting is because the patient does not think that the symptom is severe enough to report to their medical team. Patients’ decisions to report or not to report their symptoms may thus be affected by how interested they think their medical team is in patients’ efforts to report experiences with therapy. Not being sure if the sensation being experienced was a side effect of their treatment and the feeling that it was something they could handle on their own were also provided more frequently than fear of treatment being discontinued as reasons to not report symptoms.

The study results suggest that there was little influence on reporting behaviors based on culture or healthcare system differences between the USA and the European countries included in the study. The most common types of symptoms that patients experienced and did not report were gastrointestinal, respiratory, and energy related. When regrouping the patient sample into high and low social media engagers, the results were similar. Only one difference was observed for the experience of pain, indicating that high-level social media engagers reported having (but still not reporting to their medical teams) more pain than low-level engagers. This may be attributable to the support systems that virtual communities provide, including the sharing of coping mechanisms.

The findings of our study suggest actions that could be taken by investigators and medical teams to encourage greater reporting of possible side effects by patients receiving immunotherapy for NSCLC. Our results showed that patients are not always clear about what their medical teams want to know and what degree of severity should bring them back to the care setting for help. Providing patients with a more comprehensive list of side effects associated with immunotherapy at the outset of a trial or a treatment regimen could be an effective strategy to encourage patients to report their symptoms while undergoing treatment and to reach out for assistance in managing those that are too severe or worrisome. Patients should be counseled when starting treatment about what symptoms they might consider managing on their own and how best to do so. Such an approach could be instrumental in reducing anxiety in patients about their treatment.

Understanding patients’ experiences with treatment is key to developing accurate safety and tolerability profiles. Immune-related side effects that are low grade or occur late may not be captured in clinical trials, and drug manufacturers will not learn of these events unless they are reported by patients after the drug is commercially available. This is particularly relevant in long-term survivors who may take drugs to control their disease for months or years and may experience non-life-threatening side effects that, nevertheless, have a negative impact on quality of life and, potentially, treatment adherence.

Our study had several key strengths, including having recruited a relatively large sample for qualitative research. Qualitative interviews were conducted using rigorous methodologies. Concept saturation was achieved by the third of five transcript groups, suggesting that additional interviews with a population of the same characteristics would not be likely to yield any new information. Rates of inter-rater agreement indicated a very high level of consistency in the coding process and results. A limitation of all qualitative studies is their sample size. While qualitative results can provide a richness of data for understanding and interpretation, they cannot provide statistical comparisons.

In conclusion, the goal of the study was to evaluate why patients do or do not report treatment-related side effects to their medical providers. The results of our study show that there are a variety of reasons for patients to report or not to report treatment-related symptoms when they undergo immunotherapy for NSCLC, and those reasons are more often related to patients’ determination of the significance or value of the symptom than to fear of having treatment discontinued. Medical teams are in a key position to assist patients with education about what possible treatment-related symptoms to expect, ways for patients to manage these on their own, and guidance as to when to reach out for assistance. Improved reporting of treatment-related symptoms can help with understanding the patient experience with immunotherapy and potentially with finding ways to assist patients with tolerability issues and help them remain on their treatment. These study results make an important scientific and clinical contribution by drawing attention to the fact that side effects can be under-reported during immune checkpoint inhibitor therapy. These data provide support for routine symptom monitoring in clinical care as well as the addition of patient-reported outcomes to clinical trials.

## Data Availability

Datasets supporting the conclusions are included in this published article.

## Abbreviation

NSCLC: Non-small cell lung cancer
